# Gut Microbiota in Heart Failure Patients With Preserved Ejection Fraction (GUMPTION Study)

**DOI:** 10.3389/fcvm.2021.803744

**Published:** 2022-01-06

**Authors:** Ziyin Huang, Xiaofei Mei, Yufeng Jiang, Tan Chen, Yafeng Zhou

**Affiliations:** Department of Cardiology, Dushu Lake Hospital Affiliated to Soochow University, Suzhou, China

**Keywords:** heart failure with preserved ejection fraction, gut microbiota, alpha diversity, beta diversity, HFpEF-heart failure with preserved ejection fraction

## Abstract

**Introduction:** Heart failure with preserved ejection fraction (HFpEF) is associated with disrupted intestinal epithelial function, resulting from intestinal congestion. Intestinal congestion changes the morphology and permeability of the intestinal wall, and it becomes easy for the gut microbiota to change and transfer. Intervention on gut microbiota may become a new target for HFpEF treatment. However, the characteristics of gut microbiota in patients with HFpEF remain unknown. This preliminary report aims to detect the structure of gut microbiota in HFpEF patients so as to explore their characteristic changes, thereby providing a theoretical basis for future research.

**Methods:** This research recruited 30 patients diagnosed with HFpEF and 30 healthy individuals. Stool specimens of research subjects were collected separately, and the microarray analyses of gut microbiota were conducted by Illumina high-throughput DNA sequencing. The differences in gut microbiota composition, alpha diversity, and beta diversity between the two groups were finally obtained.

**Results:** The composition of gut microbiota was significantly different between the two groups. At the phylum classification level, the abundance of Synergistetes tended to be higher in the HFpEF group (*P* = 0.012). At genus classification level, the abundance of Butyricicoccus (*P* < 0.001), Sutterella (*P* = 0.004), Lachnospira (*P* = 0.003), and Ruminiclostridium (*P* = 0.009) in the HFpEF group were lower, while the abundance of Enterococcus (*P* < 0.001) and Lactobacillus (*P* = 0.005) were higher. According to the Chao index of alpha diversity analysis, HFpEF patients showed a nominally significant lower species richness when compared with controls (*P* = 0.046). However, there was no statistical difference in the Shannon index (*P* = 0.159) and Simpson index (*P* = 0.495), indicating that there was no difference in species diversity between the two groups. Beta diversity analysis revealed a highly significant separation of HFpEF patients and controls.

**Conclusions:** An imbalance in the gut microbiota of HFpEF patients was observed. Patients with HFpEF have an increased abundance of microbiota associated with inflammation and a decreased abundance of microbiota associated with anti-inflammatory effects in the gut environment. In line with that, the species richness of gut microbiota in HFpEF patients tended to be lower.

## Introduction

Heart failure with preserved ejection fraction (HFpEF) is a common cardiovascular disease with a poor prognosis (1). In spite of current medical treatment approaches, mortality of HFpEF remains high and novel treatment concepts are thus urgently required (2). A theory proposed recently showed a possible impact of gut microbiota on the incidence and progression of HFpEF (3).

The gut microbiota resides in the intestine of hosts and participates in various physiological processes of hosts. According to the classification method of biological research, gut microbiota can be classified into several types with different characteristics at seven levels, which are called a kingdom, phylum, class, order, family, genus, and species. At the phylum classification level, the gut microbiota in the human body can be divided into six main subtypes, including Bacteroidetes, Firmicutes, Proteobacteria, Actinobacteria, Fusobacteria, and Verrucomicrobia. Among them, Firmicutes and Bacteroidetes are the dominant microbiota in the healthy human body. The gut microbiota remains relatively stable. However, the changes in physiological functions can cause an imbalance of gut microbiota, which in turn can further cause a series of pathological changes in the human body. Dysbiosis of gut microbiota has been proposed to be related to several diseases including diabetes, hypertension, atherosclerosis, thrombotic events, and infectious diseases (such as tuberculosis) (4–6). For example, Luo et al. found that specific gut microbiota may be associated with the host's immune status and affect the prognosis and outcome of tuberculosis patients (7).

The gut hypothesis of heart failure (HF) is a hot spot in the field of cardiovascular research. It has been proposed that HF is associated with disrupted intestinal epithelial function, likely as a consequence of reduced intestinal perfusion. HF patients are also accompanied by intestinal congestion. Intestinal ischemia and congestion change the morphology and permeability of the intestinal wall, and the gut microbiota is prone to change and shift. The imbalance and displacement of gut microbiota further induce or aggravate systemic inflammation, which affects the progression of HF (8–12). The increase in intestinal wall permeability also makes it easier for endotoxin (such as lipopolysaccharide, LPS) to translocate to the systemic circulation. LPS levels in the blood directly correlate with systemic inflammation in HF patients (13). In addition, researchers found that there was a significant imbalance of protective metabolites and harmful metabolites in HF patients (9, 14).

Heart failure with preserved ejection fraction is a kind of heart failure characterized by diastolic dysfunction. HFpEF is associated with disrupted intestinal epithelial function, likely as a consequence of intestinal congestion, which also changes the morphology and permeability of the intestinal wall, and the gut microbiota is prone to change and shift (15–19).

However, recently published research investigating this topic is all aimed at patients diagnosed with heart failure with reduced ejection fraction (HFrEF). Studies on the gut microbiota in HFrEF patients have identified a reduction in gut microbial diversity (10). Whether the gut microbiota of HFpEF patients is different and how it contributes to the development of HFpEF are still unknown. This study aims to detect the structure of gut microbiota in HFpEF patients through high-throughput sequencing and to explore the characteristic changes of gut microbiota in HFpEF patients. We hope that the results from our research will be clinically beneficial for the treatment of HFpEF.

## Materials and Methods

### Study Population

This study has been approved by the Ethics Review Board of the First Affiliated Hospital of Soochow University and Dushu Lake Hospital Affiliated to Soochow University and is a preliminary report of study registered in the Chinese Clinical Trial Registry (ChiCTR2100050121). Research subjects were assessed for eligibility and written informed consent.

This research recruited 30 patients diagnosed with HFpEF between August 2020 and October 2021 and 30 people who underwent physical examination in the same hospital. HFpEF patients were approached during their regular clinic visits.

The inclusion criteria of the HFpEF group required compliance with the HFpEF diagnostic criteria in the “2021 ESC Guidelines for the Heart Failure” (20). The inclusion criteria of the control group included people with no cardiovascular diseases such as high blood pressure, coronary heart disease, cardiomyopathy, pericardial disease, and valvular heart disease. At the same time, according to the diagnostic criteria of heart failure, heart failure should be excluded based on clinical assessment and NT-proBNP serum levels. The exclusion criteria for research subjects were as follows: (1) patients with acute heart failure, acute pericarditis, acute myocarditis, and congenital heart disease; (2) patients who take antibiotics orally or intravenously within three months; (3) patients using antibiotics on the skin within one month; (4) patients who ingest probiotics within three months; (5) patients with intestinal diseases (such as inflammatory bowel diseases) or patients with a history of bowel surgery and (6) patients with other non-cardiovascular diseases that have been known to change the gut microbiota.

### Sample Collection and Data Recording

Research subjects emptied their stool in special stool collection containers. After the defecation was completed, research subjects scooped up 5 g deep stool and placed it in the professional stool DNA preservation solution. The stool samples were stored at −80°C until further processing.

Baseline descriptive data such as age, gender, body mass index (BMI), New York Heart Association (NYHA) classification, main diagnosis, and past medical history of the two groups were available at the inception of the study. Laboratory tests such as blood routine, liver function, kidney function, and blood lipids were carried out within 24 h. Echocardiographic data of HFpEF patients such as left ventricular ejection fraction (LVEF) were also recorded. Clinical characteristics are presented in Table 1.

**Table 1:** Baseline descriptive data of two groups.

	**HFpEF group (*n* = 30)**	**Control group (*n* = 30)**	***P*** **value**
**Male (** ***n*** **, %)**	19 (63.33%)	17 (56.67%)	0.598
**Age (year)**	71.20 ± 9.36	67.03 ± 7.43	0.061
**BMI (kg/m** ^**2**^ **)**	23.83 ± 3.04	23.85 ± 2.95	0.984
**NYHA**			
I	0	–	–
II	6 (20%)	–	–
III	18 (60%)	–	–
IV	6 (20%)	–	–
**Medical history**			
CHD	15 (50%)	–	–
Hypertension	25 (83.33%)	–	–
Hypertrophic cardiomyopathy	6 (20%)	–	–
**Laboratory tests**			
WBC (10^∧^9/L)	6.00 ± 2.10	5.76 ± 1.14	0.591
PLT (10^∧^9/L)	159.73 ± 49.90	194.20 ± 50.59	**0.010**
HB (g/L)	132.93 ± 12.92	136.63 ± 17.60	0.357
ALT (U/L)	17.35 (12.78, 32.65)	20.40 (13.08, 25.98)	**0.011**
AST (U/L)	25.45 (18.08, 31.80)	21.10 (16.78, 25.30)	0.929
Cr (umol/L)	72.90 (62.20, 85.03)	59.40 (51.13, 72.43)	0.090
TG (mmol/L)	1.38 ± 0.72	1.28 ± 0.74	0.626
TC (mmol/L)	3.92 ± 0.98	4.73 ± 1.11	**0.004**
LDL-C (mmol/L)	1.05 ± 0.33	1.27 ± 0.28	**0.008**
HDL-C (mmol/L)	2.28 ± 0.76	2.74 ± 0.88	**0.037**

*HFpEF, Heart failure with preserved ejection fraction; BMI, Body Mass Index; NYHA, New York Heart Association*.

### Sample Detection

The DNA of gut microbiota was extracted by using the QIAamp DNA stool kit. We designed the target region and fusion primers according to the requirements of the sequencing platform and used a two-step method for PCR amplification. Variable regions V4–V5 of the 16S rRNA gene were amplified. The PCR amplification product obtained in the previous step was recovered by using 2% agarose gel. We used the AxyPrep DNA Gel Extraction Kit to purify the amplified PCR product. Finally, we used the Illumina sequencing platform to perform high-throughput sequencing on the samples that we collected.

### Data Analysis

The differences in gut microbiota between the two groups were mainly presented in three aspects, which included the composition of gut microbiota, alpha diversity, and beta diversity. The composition of gut microbiota between the two groups was presented at the classification level of phylum and genus. Wilcoxon rank sum test was used to compare the abundance of each microbiota when the value distribution deviated from normality, while two samples *t*-test was used when there was no deviation. Rank-abundance distribution curves and VEEN picture based on an operational taxonomic unit (OTU) were also used to compare the composition of gut microbiota between the two groups. When testing the differences in alpha diversity, the Wilcoxon rank sum test was used to compare three indexes (Chao index, Shannon index, and Simpson index) between the two groups when the value distribution deviated from normality. The principal co-ordinates analysis (PCoA) chart based on Bray-Curtis was used to present the results of beta diversity analysis, and Anosim analysis was carried out further. We also performed a subgroup analysis of the HFpEF group according to their etiologies. Alpha diversity analysis and beta diversity analysis were calculated by using R.

## Results

### Composition of gut Microbiota in HFpEF Patients and Controls

This study mainly analyzed the composition of gut microbiota at the classification levels of phylum and genus and compared the differences in gut microbiota composition between the two groups.

**Figure 1:**
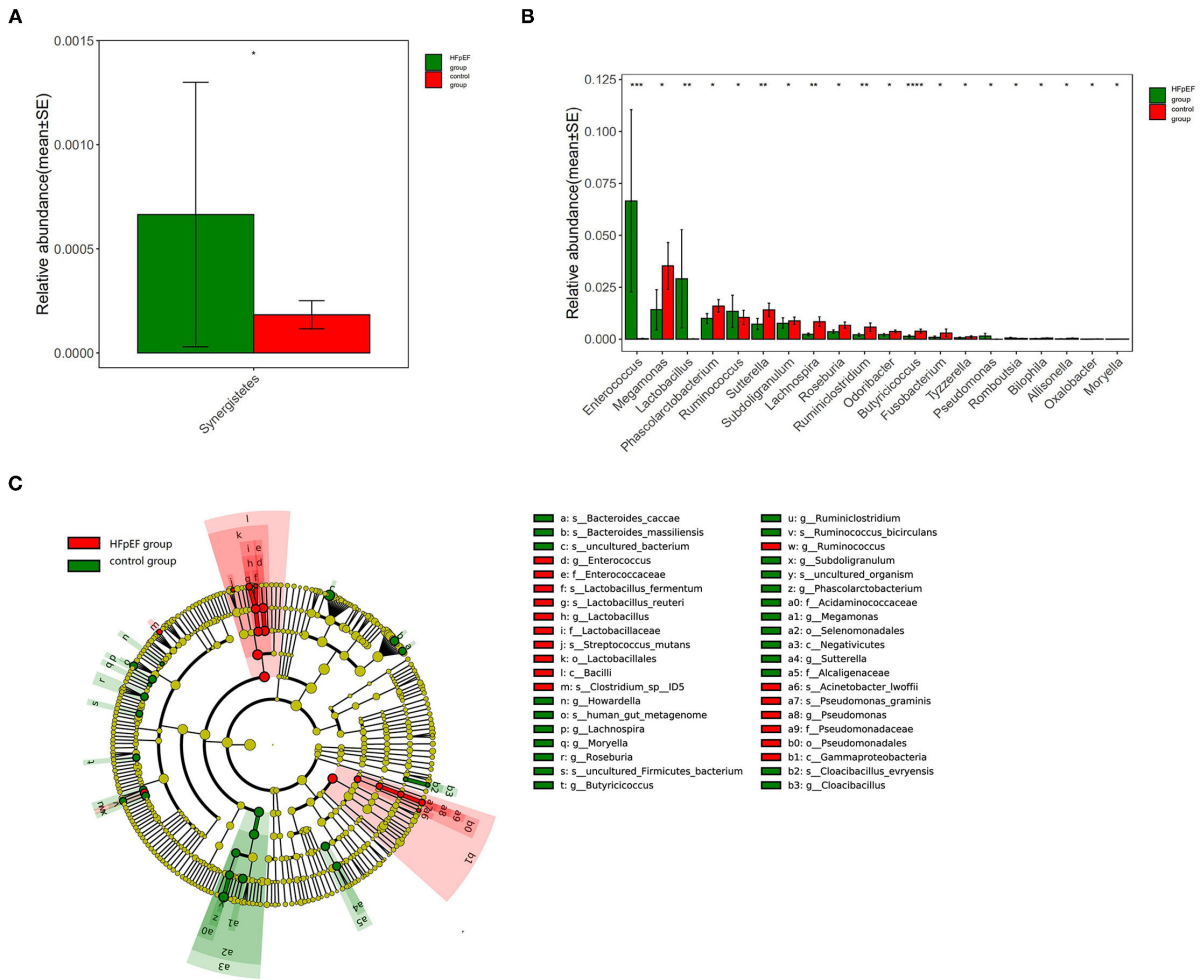
Differences in gut microbiota composition in patients with Heart failure with preserved ejection fraction (HFpEF) and controls. **(A)** Differences in gut microbiota composition at phylum classification level. **(B)** Differences in gut microbiota composition at genus classification level. **(C)** The results of linear discriminant analysis effect size (LEfSe) analysis. ^*^0.01 < *p* < 0.05, ^**^0.001 < *p* < 0.01, ^***^0.0001 < *p* < 0.001, ^***^p < 0.0001.

Relative abundance refers to the proportion of one microbiota in the total microbiota (i.e., the sequences number of one microbiota divided by the total sequences number). The result indicated that the HFpEF group mainly included Bacteroides, Firmicutes, Proteobacteria, Actinobacteria, Fusobacteria, Tenerictes, Verrucomicrobia, Synergetes, Lentisphaerae, Cyanobacteria, Spirochaeta, and Unclassified microbiota. On the other hand, the control group consisted of Bacteroides, Firmicutes, Proteobacteria, Actinobacteria, Fusobacteria, Tenerictes, Verrucomicrobia, Synergistetes, Lentisphaerae, and Unclassified. The microbiota with the highest abundance in HFpEF patients and controls were Bacteroides, Firmicutes, and Proteobacteria. The microbiota with a statistically significant difference at the phylum classification level was Synergistetes (*P* = 0.012). In other words, the abundance of Synergistetes tended to be higher in the HFpEF group when compared with the control group (Figure 1A).

At the genus classification level, Bacteroides and Prevotella were two kinds of microbiota with the highest abundance. The abundance of Butyricicoccus (*P* < 0.001), Sutterella (*P* = 0.004), Lachnospira (*P* = 0.003), and Ruminiclostridium (P=0.009) in the HFpEF group were lower than that in the control group, while the abundance of Enterococcus (*P* < 0.001) and Lactobacillus (*P* = 0.005) were higher (Figure 1B).

The results of linear discriminant analysis effect size (LEfSe) analysis showed that the characteristic microbiota in the HFpEF group was Enterococcus, Lactobacillus, et al., while in the control group were Sutterella, Lachnospira, Ruminiclostridium, Butyricicoccus, et al. (Figure 1C).

### Rank-Abundance Distribution Curves and VEEN Picture Based on OTUs

The detected sequences in Illumina high-throughput DNA sequencing can be classified according to a certain similarity. Operational taxonomic unit (OTU) refers to the sequences grouped by a certain similarity. We set 97% as the critical value, that is, if the sequence similarity ≥ 97%, it can be divided into the same OTU.

**Figure 2:**
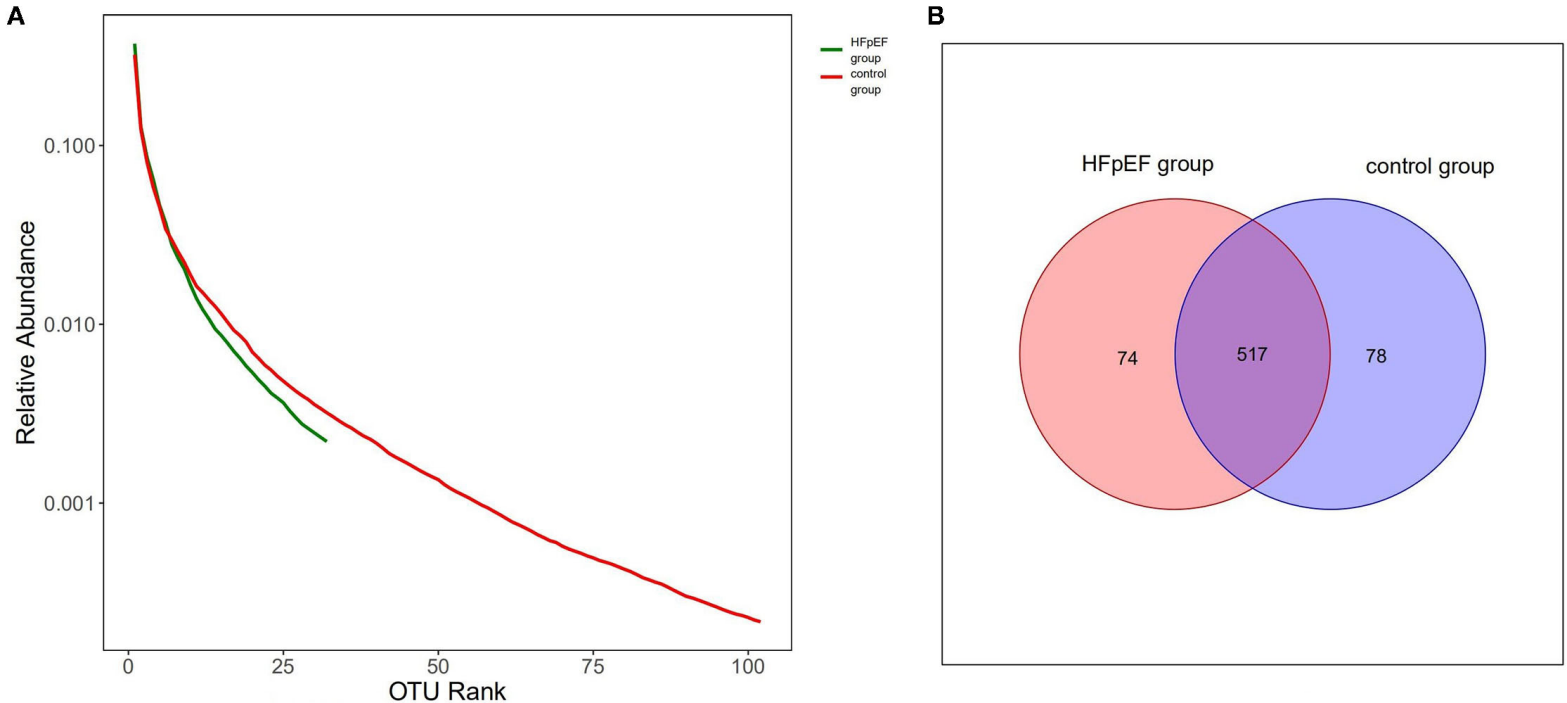
Rank-abundance distribution curves and VEEN picture based on operational taxonomic units (OTUs). **(A)** Rank-abundance distribution curves based on OTUs. **(B)** VEEN picture based on OTUs.

We drew Rank-abundance distribution curves and VEEN picture based on OTUs. The ordinate of Rank-abundance distribution curves represents the relative abundance of each OTU and the abscissa is the OTU arranged from high to low. The width of the curve reflects the species richness, while the shape of the curve can be used to explain the species evenness. As shown in Figure 2A, the species richness and evenness of the HFpEF group were lower than that of the control group. According to Figure 2B, the total number of OTUs in the HFpEF group was lower.

### Differences Between HFpEF Patients and Controls With Regard to Alpha and Beta Diversity

**Figure 3:**
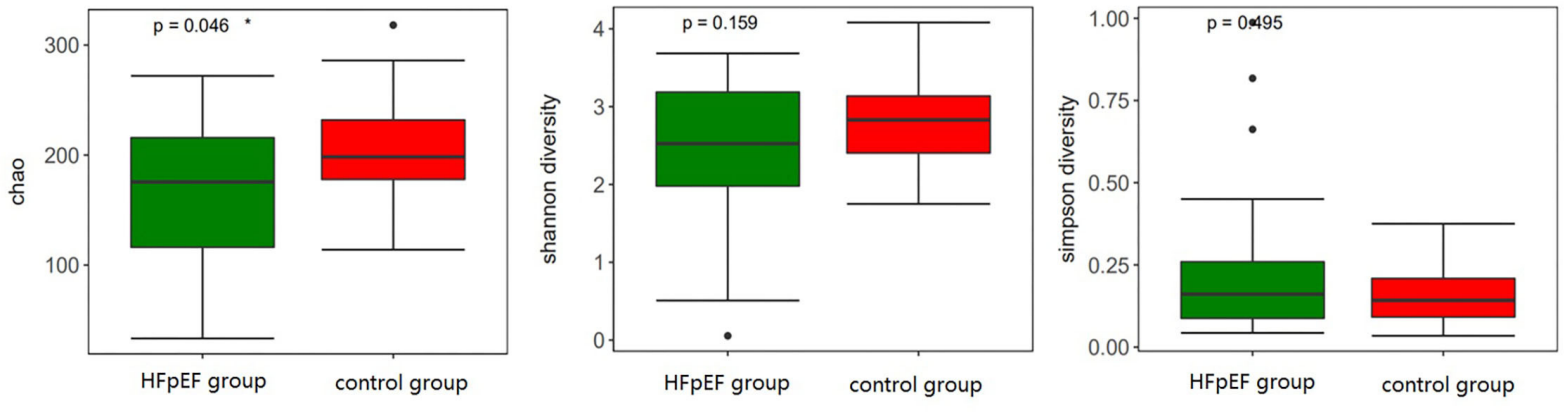
Differences in alpha diversity between patients with HFpEF and controls ^*^0.01 < *p* < 0.05.

With regard to alpha diversity, which reflected intra-individual variance, three indexes (Chao index, Shannon index, and Simpson index) were evaluated. There was a difference in the Chao index between the two groups (*P* = 0.046), which meant that the species richness of gut microbiota in the HFpEF group was lower than that in the control group. However, there was no statistical difference in the Shannon index (*P* = 0.159) and Simpson index (*P* = 0.495), indicating that there was no difference in the species diversity between the two groups (Figure 3).

**Figure 4:**
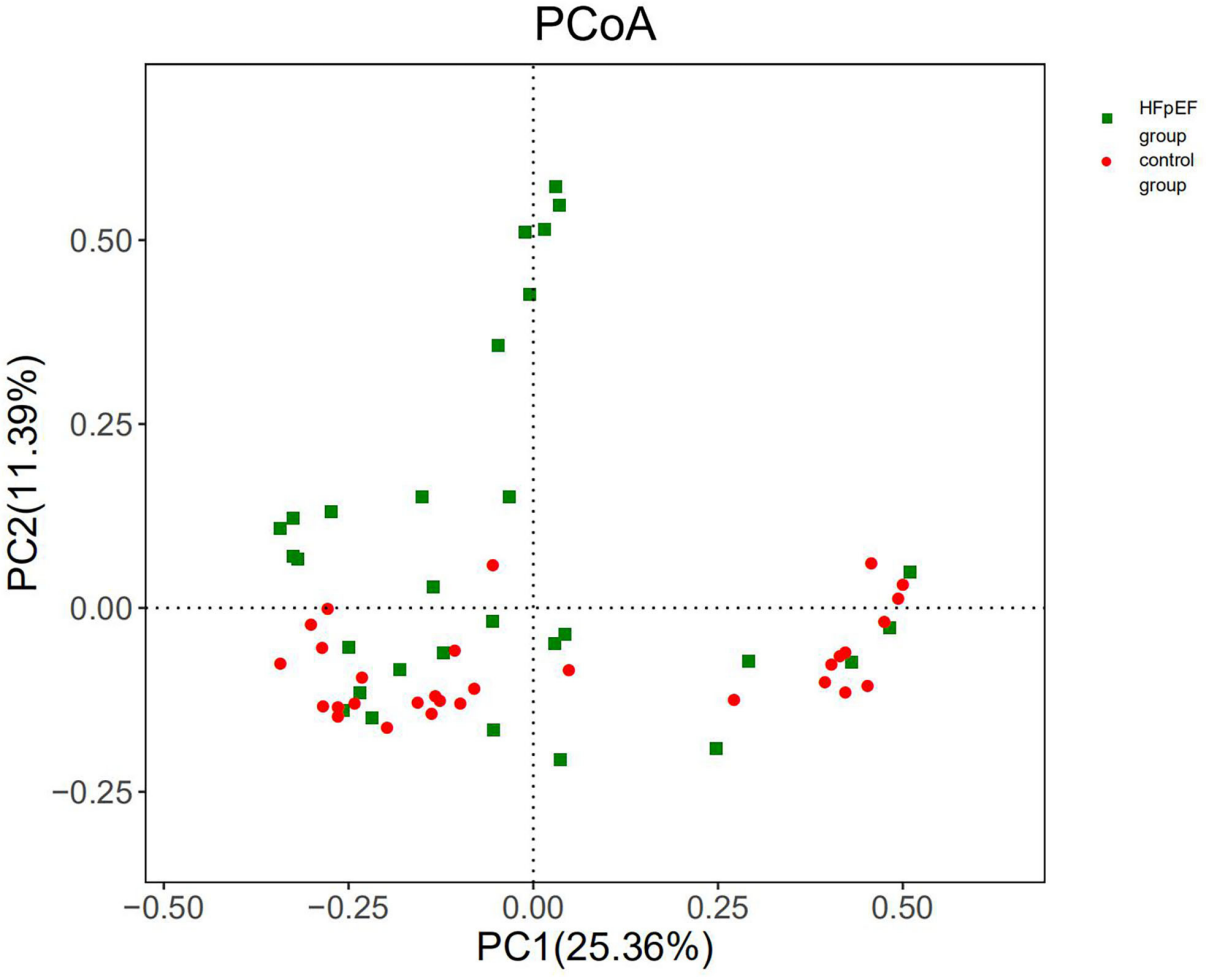
Beta diversity differed significantly in patients with HFpEF and controls. PC1, principal component 1; PC2, principal component 2.

Assessment of beta diversity, a parameter that represents inter-individual variances, showed a significant separation of HFpEF patients and controls (*P* < 0.05). The results provided evidence for a shift in the composition of gut microbiota induced by HFpEF. The PcoA chart based on Bray-Curtis was used to present the results of beta diversity analysis (Figure 4). Anosim analysis of this study showed that the differences between the groups were greater than the differences within the groups (*R* = 0.051, *P* = 0.028).

### Differences Between HFpEF Patients With Different Etiologies

We divided 30 HFpEF patients into three subgroups according to their etiologies: hypertensive heart disease group, coronary heart disease (CHD) group, and hypertrophic cardiomyopathy group. At the phylum classification level, there was no significant difference in the abundance of each microbiota. However, at the genus classification level, the abundance of 10 microbiota, such as Weissella (*P* = 0.008), Selenomonas (*P* = 0.006), and Dialister (*P* = 0.007), was statistically different between HFpEF patients, which was caused by different etiologies. In alpha diversity analysis, the Shannon index (*P* = 0.025) and Simpson index (*P* = 0.016) were statistically different, which indicated that the species diversity of gut microbiota in the CHD group was higher than that in the hypertensive heart disease group and hypertrophic cardiomyopathy group (*P* = 0.036, *P* = 0.011). To sum up, the composition and species diversity of gut microbiota was also different between HFpEF patients, which was caused by different etiologies.

## Discussion

In this research, we determined the differences in gut microbiota between HFpEF patients and controls. At the phylum classification level, the abundance of Synergistetes in the HFpEF group tended to be higher. At the genus classification level, the abundance of Butyricicoccus, Sutterella, Lachnospira, and Ruminiclostridium in the HFpEF group was lower than that in the control group, while the abundance of Enterococcus and Lactobacillus was higher. Enterococcus belongs to a class of conditional pathogenic microbiota in the human gut. *In vitro* studies confirmed that Enterococcus can generate a kind of medium related to the inflammatory response, namely polymorphonuclear leukocyte chemotactic factor (PCF). In addition, clinical research demonstrated that Enterococcus is associated with endocarditis and urinary tract infections (21–23). Lactobacillus can decompose glucose and other sugars into lactic acid. An animal experiment found that Lactobacillus can aggravate the systemic inflammatory response in animals (24, 25). At the same time, patients with HFpEF had a depletion of microbiota known to be associated with anti-inflammatory effects. Butyricicoccus, Sutterella, Lachnospira, and Ruminiclostridium were all found to be related to anti-inflammatory effects *in vitro* studies or animal studies. For instance, Butyricicoccus can change the energy source of intestinal cells from glycolysis to fatty acid metabolism. A large amount of butyric acid will be produced in the process of fatty acid metabolism, which can downregulate the concentration of proinflammatory mediators and reduce inflammation in the human body (26–29). To sum up, the changes in gut microbiota in HFpEF patients included an increase in the abundance of microbiota associated with inflammation and a decrease in the abundance of microbiota associated with anti-inflammatory effects. This prolonged inflammatory state induced or aggravated by the gut microbiota imbalance may have a bad impact on the prognosis of patients with HFpEF. More research should focus on the mechanisms involving altered inflammatory pathways induced by gut microbiota imbalance. At the same time, maintaining the stability of gut microbiota in HFpEF patients through diet adjustment, antibiotic use, probiotic preparation use, fecal bacteria transplantation, and other methods is expected to become a new target of HFpEF treatment (30–32).

With regard to alpha diversity, our data pointed out that the species richness of gut microbiota in the HFpEF group was lower than that in the control group. Consistent with this, the results of Rank-abundance distribution curves based on OTU indicated that the species richness and species evenness of gut microbiota in the HFpEF group tended to be lower. Patients with HFpEF had a depletion of microbiota associated with anti-inflammatory effects, which was a driver for the reduction in gut microbial richness. Studies on the gut microbiota in HFrEF patients have also identified a reduction in gut microbial richness. The decrease in species richness had a bad impact on the prognosis of HFrEF patients (33–35). Therefore, we speculate that the reduction in species richness may also have an adverse effect on HFpEF patients. Beta diversity showed a separation of HFpEF patients and controls. Anosim analysis showed that the difference between the HFpEF group and the control group was greater than that within the group.

The general opinion favors the concept that gut microbial imbalance arises as a consequence of cardiac dysfunction. However, an impaired gut microbiota as a disease marker for the progress of HFpEF seems to be conceivable as well. Future studies could assess if alterations in disease status during the progression of HFpEF are mirrored by alterations in gut microbial composition (36–38).

This study provided valuable information on the characteristic changes of gut microbiota in HFpEF patients. Our data showed that an altered intestinal microbiome might be a potential player in the pathogenesis and progression of HFpEF. Moreover, we hope that the results from this research will be clinically beneficial for the specific treatment of HFpEF.

## Conclusion

There was an imbalance of gut microbiota in HFpEF patients. Patients with HFpEF have an increased abundance of microbiota associated with inflammation and a decreased abundance of microbiota associated with anti-inflammatory effects in the gut environment. Moreover, the species richness of gut microbiota in HFpEF patients tended to be lower. We hope that the findings of this study will provide new directions for the clinical treatment of HFpEF.

## Data Availability Statement

The datasets presented in this study can be found in online repositories. The names of the repository/repositories and accession number(s) can be found below: NCBI with BioProject ID PRJNA786060 (https://dataview.ncbi.nlm.nih.gov/object/PRJNA786060?reviewer=ibvi0m4rj6766p3qbdsspnl567).

## Ethics Statement

The studies involving human participants were reviewed and approved by Suzhou Dushu Lake Hospital Ethics Board. The patients/participants provided their written informed consent to participate in this study. Written informed consent was obtained from the individual(s) for the publication of any potentially identifiable images or data included in this article.

## Author Contributions

ZH, YJ, and YZ designed the study. ZH and XM wrote the first draft of this report. YZ, TC, and YJ helped to write the final version. All authors read and met the criteria for authorship and agree with the results and conclusions of the report.

## Funding

This work was supported by grants from the National Natural Science Foundation of China (81873486), Suzhou Promoting Health through Science and Education Youth Science and Technology Project (KJXW2020001), Medical research project of Jiangsu Provincial Health Commission (Z2021011), Biomedical translational medicine innovation and applied research project (SZM2021020) and Project of Suzhou Science and Technology Development Program (Applied Basic Research, SKJYD2021045).

## Conflict of Interest

The authors declare that the research was conducted in the absence of any commercial or financial relationships that could be construed as a potential conflict of interest.

## Publisher's Note

All claims expressed in this article are solely those of the authors and do not necessarily represent those of their affiliated organizations, or those of the publisher, the editors and the reviewers. Any product that may be evaluated in this article, or claim that may be made by its manufacturer, is not guaranteed or endorsed by the publisher.
